# Use of normalization methods for analysis of microarrays containing a high degree of gene effects

**DOI:** 10.1186/1471-2105-9-505

**Published:** 2008-11-28

**Authors:** Terri T Ni, William J Lemon, Yu Shyr, Tao P Zhong

**Affiliations:** 1 Division of Cardiovascular Medicine, Department of Medicine, Vanderbilt University School of Medicine, Nashville, TN 37232, USA; 2 Division of Cancer Biostatistics, Department of Biostatistics, Vanderbilt University School of Medicine, Nashville, TN 37232, USA; 3 Department of Statistics, National Cheng Kung University, Taiwan, ROC; 4 Academic Programs, Missouri Tech, St. Louis, MO 63132, USA

## Abstract

**Background:**

High-throughput microarrays are widely used to study gene expression across tissues and developmental stages. Analysis of gene expression data is challenging in these experiments due to the presence of significant percentages of differentially expressed genes (DEG) observed between tissues and developmental stages. Data normalization methods that are widely used today are not designed for data with a large proportion of tissue or gene effects.

**Results:**

In our current study, we describe a novel two-dimensional nonparametric normalization method for analyzing microarray data which functions well in the absence or presence of large numbers of gene effects. Rather than relying on an assumption of low variability among most genes, the method implements a unique peak selection strategy to distinguish DEG from genes that are invariant in expression, prior to nonlinear curve fitting. We compared the method under simulated and experimental conditions with five alternative nonlinear normalization approaches: quantile, lowess, robust lowess, invariant set, and cross-correlation (Xcorr). Simulations included various percentages of simulated DEG and the experimental data used is from publicly available datasets known to be difficult to analyze due to the presence of approximately 34% DEG.

**Conclusion:**

We have demonstrated that the new method provides considerable improvement in the accuracy of data normalization when large proportions of gene effects are present. The performance improvement is mostly attributed to its variable selection component, which is designed to separate expression invariant genes from DEG. Adding this key component of the new method to alternative normalization approaches rescues the most of the sensitivity of these methods to gene effects. The results indicate that our method may be used without prior knowledge of or assumptions about housekeeping genes to normalize microarrays that are quite different.

## Background

Identifying microarray target concentration changes in response to developmental and environmental cues is one of the most common end points of microarray data analysis. However, diverse and numerous sources of variation are known to affect the accuracy and reliability of such results [1,2]. Large community-wide efforts including The Microarray Quality Control (MAQC) Project [3] and The External RNA Controls Consortium (ERCC) [4] have been initiated to further identify sources of variation, establish reliable assessment metrics, and improve the accuracy of the interpretation of microarray data. Normalization is an important analytical step to correct systematic noise inherent in microarray technology. To make arrays comparable current normalization techniques rely on knowledge or assumptions about genes expected to have low variation. We present a novel microarray normalization method that avoids the need to know or hypothesize about genes with low variation and thereby provide a more robust procedure.

Not all sources of variation that are known to have an impact on data normalization performance are noise. Biological sources of variation (gene effects), namely, differential gene expression, are frequently observed in microarray data. In raw measurements, it is difficult to distinguish between biological sources of variation and the variation due to limited sampling, differences in array production batches, hybridization and washing conditions, scanning power, etc. When arrays under comparison are generated from different tissues or developmental stages, the total impact of biological variation and the percentage of genes containing gene effects are expected to be significantly large. This presents a challenge to microarray data normalization, which is an important step for removing experimental noise. A salient question with respect to normalization is how to deconvolute two sources of variation so that the associated experimental noise can be accurately assessed and removed by normalization. With proper normalization, the underlying biological variation across experimental groups becomes quantifiable.

Frequent observation of nonlinear systematic noise in both Affymetrix chip data and cDNA microarrays has led to the development of several normalization methods for correcting nonlinear experimental differences between arrays. Among them, quantile normalization in RMA [5], lowess [6], and invariant set method [7,8] are well adopted for analyzing Affymetrix chip data. To prevent the perturbation caused by the presence of gene effects or other sources of outliers, lowess provides a built-in robust regression option in the method (robust lowess). Designed for the same purpose, the invariant set method selects expression invariant probes whose rank difference is proportionally small between two chips prior to nonlinear curve fitting via either Lowess [8] or median-based procedures [7]. These approaches provide robustness against small gene effects.

However, they require that a critical assumption hold: any gene effects that exist are small or symmetric over the entire intensity range [9]. Therefore, dChip recommends users to provide a known set of housekeeping genes which are assumed to have low variability across experimental groups when normalizing arrays from different tissues. To relax the statistical assumption, Fan *et al*. proposed a semilinear in-slide model to iteratively assess two sources of variation [10,11] and thereby directly address the issue of gene effects. Variations of the semilinear models have also been proposed [12]. These methods often impose the requirement that sufficient numbers of replicated genes are present over the entire intensity range. With all these methods, there is a demand for a set of known low-variability genes over the intensity range.

Performance of various normalization methods have been evaluated and compared in many studies. Most evaluation does not involve data with significantly large numbers of gene effects, due to a lack of suitable experimental microarrays. Recently, Fujita et al compared several nonlinear normalization methods including lowess using an artificial dataset containing up to 40% outliers [13]. Their results indicate various degrees of robustness to outliers with respect to normalization choices. When the percentage of outliers increases from 5% up to about 40%, all methods incur increasing amounts of normalization errors. To overcome the lack of experimental microarrays of known large gene effects, Choe et al generated a spike-in array dataset (named Golden Spike) containing approximately 34% gene effects [14]. As expected, normalization based on only unchanged genes clearly performs better than normalization on both unchanged genes and differentially expressed genes (DEG), indicating that the deleterious impacts of incorrect assumptions about which genes have no gene effects are present in all normalization methods studied. In light of this study, Irizarry et al. stress the need for new normalization methods when processing microarray datasets that are quite different [15].

In our current study, we propose a two-dimensional nonparametric modeling approach named nonparametric variable selection and approximation (NVSA) for normalizing Affymetrix arrays in experiments that may have significantly large numbers of gene effects. NVSA identifies genes exhibiting no differential expression, and uses them as the basis for normalization. Using benchmark evaluation procedures, we demonstrate the following: 1) NVSA prevents interference by gene effects and results in higher accuracy and higher precision normalization under conditions where the performance of alternative approaches are affected; 2) the variable selection component of NVSA can be used to improve the performance of alternative methods; and 3) NVSA analysis generates statistically consistent data whether all genes or only housekeeping genes are used for normalization. Taken together, our data indicates that NVSA may be a useful utility for data normalization analysis in any experiment, and especially in experiments currently known or expected to be difficult to analyze such as those containing samples from different tissues or development stages.

## Results

### The NVSA methodology

Let *x*_*i*_, *y*_*i*_, *i *= 1, 2,... *n *be the measured log_2 _baseline and sample array intensity at the *i*th gene, or probe, respectively. Following the MA convention [6], we thus have

(1) *M*_*i *_= *y*_*i *_- *x*_*i *_= *δ*_*i *_+ *f*(*A*_*i*_) + *ε*_*i*_

where *δ*_*i *_denotes gene effect, *A*_*i *_= 0.5* (*x*_*i *_+ *y*_*i*_), *f*(*A*_*i*_) is the intensity effect and *ε*_*i *_is random error. To estimate the intensity and gene effect levels independently, we first fixed *f*(*A*_*i*_) by segmentation or binning. To provide sufficient degrees of freedom to estimate *f*(*A*_*i*_), the data were binned into Q fixed-width intervals by *A*. When the width is sufficiently small, the *A*within an interval will be similar, thus the corresponding *f*(*A*_*i*_) is approximately constant. Then for the interval J centered at *A*_*j*_, (1) is rewritten as

(2) *M*_*j *_= *δ*_*j *_+ *f*(*A*_*j*_) + *ε*_*j*_

where *A*_*j *_∈ *J*, *j *= 1, 2,...,*m*. This equation indicates that only when the gene effect *δ *= 0, can the intensity effect *f*(*A*_*j*_) be solved independently from the gene effect. Three classes of gene effect may exist: no gene-regulation (*δ *= 0), up-regulation (*δ *> 0), and down-regulation (*δ *< 0). Therefore, the density distribution of {*M*_*j*_} may be skewed, as well as multimodal, depending on the relative proportions of these three gene effect classes.

Let *r*_*j *_= *M*_*j*_, *f*(*r*) be the density of *r *with *k *numbers of distinct classes or modes, *k *>= 1. We approximate *f*(*r*) via one-dimensional Gaussian kernel density estimation [16]:

(3) $$\hat{f}(r;h)=\frac{1}{mh}\sum_{J=1}^{M}k(r-r_{j})$$

(4) $$K(u)=\frac{1}{\sqrt{2\pi }}e^{-u^{2}/2}$$

where *K*(*u*) is a Gaussian with zero mean and standard deviation of 1 and *h *is the fixed width smoothing window (h = 0.125, half of the bin width). The boundaries of each mode (class) are identified via gradient search:

(5) $$grad(grad(\hat{f}(r)))\geq 1$$

where *grad*($\hat{f}$(*r*)) ≥ 0.001. The expected value of each class ∏_*i*_, *i *= 1, 2,...,*k *is computed by averaging the top 80% of the peak area of the class to prevent convolution between classes. We are primarily interested in identifying the invariant class ∏^*iv*^, which is enabled by following the two priors: 1) predominance rule: The area in ∏^*iv *^is the largest when invariant genes are dominant, and 2) intensity effect *f *(*A*) is slowly changing [17] along *A*, as does the expected value of the invariant class (*E*(∏^*iv*^)). Let ∏^*L *^be the class with largest area, *w*^*L *^be the ratio of the largest class to the total area in all classes within an interval. We compute the central moving standard deviation (MSD) on *w*^*L *^data and moving coefficient of variation (MCV) on *E*(∏^*L*^) data, respectively, across intervals with window size of 3. The initial seed invariant classes were selected once relative homogeneity in predominant classes occurs (MSD <= 0.2 and MCV <= 0.2). The invariant classes for the remaining intervals are determined sequentially from the seed to the left boundary and then from the seed to the right, employing the slow-changing rule:

(6) arg min {|*f'*(*x*)|} ∩ arg min {|*f"*(*x*)|}

where $f^{\prime }(x_{i})=E(\prod_{i})-E(\prod_{-1}^{iv})$, $f^{\prime }(x_{-1})=0.5\times ((E(\prod_{-1}^{iv})-E(\prod_{-2}^{iv})+f^{\prime }(x_{-2}))$, *f"*(*x*_*i*_) = *f'*(*x*_*i*_) - *f'*(*x*_-1_), -1, -2 denote the preceding two intervals, respectively. If none of the classes in the interval to be evaluated meets the condition specified in equation [6], the interval will be skipped. The intra-bin intensity effect is then derived by *f*(*A*_*J*_) = *E*(∏^*iv*^), according to [2]. To compute the fitted intensity effects {$\hat{f}$(*A*_*i*_)}, Q numbers of bin-conditioned intensity effects {*f*(*A*_*J *_}) are smoothed on intensities {*A*_*J*_} by a weighted smoothing spline [18], where weight is defined as:

(7) $$w(n)=\frac{n-n_{\min}}{n_{\max}-n_{\min}}\times (\tau_{a}-\tau_{b})+\tau_{b}$$

where *n *is the logarithm to the base 2 of the number of data points in an interval, *τ*_*a*_, *τ*_*b *_are tolerance factors of a smoothing spline, and *τ*_*a *_≤ *τ*_*b *_(default values *τ*_*a *_= 0.01 and *τ*_*b *_= 0.05). The final normalized log_2 _intensity is given by, ${y^{\prime }}_{i}=y_{i}-\hat{f}(A_{i})$. The NVSA was written in MatLab [19] and is available free of charge for academic purposes upon request.

### Normalization accuracy and precision under conditions of gene effects

**Figure 1:**
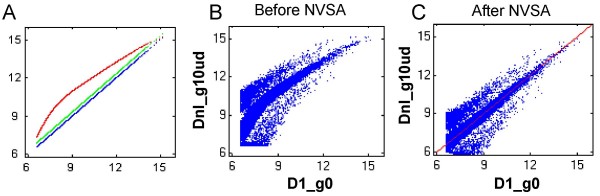
**Removal of nonlinear intensity effects by NVSA**. (*A*) Simulated scaling factor as a function of intensity in the constructed simulation data series D1 (blue), D1.25 (green), and Dnl (red). Gene and random effects are not introduced so that the scaling factor line can be revealed. The log_2 _true intensities (x-axis) and the corresponding log_2 _(true intensities × scaling factors) are plotted. (*B-C*) Typical intensity scatter plots illustrating the nonlinearity before normalization (*B*) and removal of nonlinearity after (*C*) NVSA normalization. D1_g0 data (x-axis) is constructed using a linear scaling factor of 1 (blue in A) and zero gene effects plus random effects. Dnl_g10ud (y-axis) is simulated with the nonlinear scaling factors shown in *A *(red), 5% up-regulated plus 5% down- regulated genes (a total of 10% gene effects) in addition to random effects. Each blue dot represents a feature on the simulated microarray. The red line is 45-degree reference line.

To evaluate whether our NVSA method would be sufficiently robust against the impact of gene effects, we constructed simulated data based on a simple multiplicative error model with the ground truth data generated from real microarray data (Methods). Three intensity effects, three gene regulation modes, and 12 various percentages of gene effects are simulated alone or in combinations. Data series labeled D1, D1.25, and Dnl represent linear intensity effects of 1 and 1.25, respectively, and nl denotes nonlinear intensity effects (Fig. 1A). Gene regulation modes are up-regulation only, down-regulation only, and mixed up- and down-regulation (1:1 ratio) known as a globally symmetric gene effect.

Six alternative normalization procedures, quantile, lowess, robust lowess, invariant set, cross-correlation (Xcorr), and global median were chosen to be compared with NVSA due to their availability. Cross-correlation is a novel peak-matching algorithm to address unbalanced shifts in transcripts levels [20]. All datasets were normalized to the ground truth data under default conditions. Figure 1B, C shows a typical scatter plot of intensities before and after NVSA normalization, illustrating that nonlinearity is removed by NVSA normalization.

We then used accuracy and precision measurements to quantify the performance of each normalization procedure. Accuracy here refers to closeness to the true value, computed as the mean relative error in the estimated normalization factors: average of |(estimated – true)/true|. A highly accurate method incurs low error. The precision benchmark, an indication of reproducibility, refers to how near the values of repeated measurements are to each other. The array-averaged Coefficient of Variation (CV) was used to evaluate the precision between the normalized Dnl and their gene effect and regulation mode matched D1 array intensities (e.g., Dnl-10% up vs. D1-10% up regulation).

**Figure 2:**
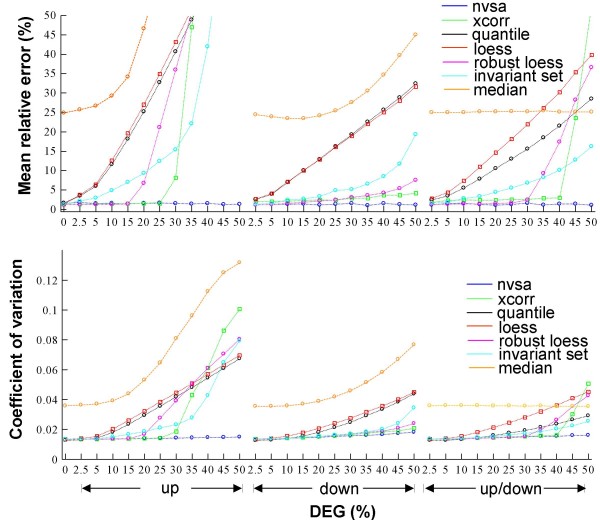
**Comparison of normalization accuracy and precision using seven different analytical methods**. (*A*) Mean relative error in normalization (scaling) factors determined by the seven different normalization methods using Dnl dataset. The relative error for each feature of the microarray is defined as |(estimated – truth)/truth|, where the mean relative error is derived by averaging all features in an array. Errors that are greater than 50% are not shown. (*B*) Coefficient of variation (CV) between D1 and gene-effect matched normalized Dnl data. D1 and Dnl are defined in Fig. 1. The x-axis in (*A-B*) corresponds to the total percentage of gene effects (shown as DEG) in a microarray, and up, down, and up/down indicates these effects as up-, down-, and an equal mixture of both, respectively. Xcorr: cross-correlation. Median: global median normalization.

The accuracy results show that all nonlinear normalization procedures perform equally well in conditions where there is a zero percentage of DEG (Fig. 2A). However, the accuracy of the quantile, lowess and invariant set are progressively affected by an increase in the percentage of gene effects. Robust lowess, on the other hand, appears to provide protection against gene effects ranging up to 15%. Among alternative methods, cross-correlation is most robust, exhibiting a similar low error rate until 30% gene effects. Among all methods evaluated, the errors in the NVSA-normalized data were found to be the lowest and do not appear to be significantly affected up to 50% DEG. This indicates that under gene effect conditions, NVSA is the most accurate method and is the most resistant to the presence of DEG. The accuracy results with the linear D1.25 dataset also show similar pattern [See Figure S1 of additional file 1], indicating that the performance difference in normalization is not due to the ability to remove the data nonlinearity itself, but owing to each method's robustness against gene effects.

Consistent with the differences in the data modeling strategies, the invariant set and robust lowess approaches are more robust against gene effects when compared to quantile and lowess. The improved accuracy in NVSA over the quantile and lowess is statistically significant under all 33 non-zero gene effect conditions (one-tailed paired Student's t-test *P*-value < 0.001), Likewise, the larger normalization errors in invariant set, robust loess, and cross-correlation methods than the errors in NVSA are statistically significant under 19, 10, and 9 out of a total of 33 non-zero gene effects conditions, respectively.

The precision of the normalization was measured by the array-averaged Coefficient of Variation (CV) between the normalized Dnl and their gene-effect matched D1 array intensities (e.g., Dnl-10% up vs. D1-10% up). The results (Fig. 2B) show a similar pattern to the accuracy assessment: 1) all six nonlinear normalization approaches perform equally well under zero gene effects, 2) The precision of NVSA normalization does not appear to be negatively affected by gene effects, but the precision of quantile, and lowess methods worsens when the gene effect increases, and 3) the cross-correlation, invariant set, and robust lowess approaches generally perform better than quantile and lowess under nonzero gene effect conditions. Taken together, our studies indicate that under our test conditions, NVSA normalization is of high-accuracy and high-precision regardless of the extent of the gene effects.

### NVSA improves the robustness of alternative methods against gene effects

We postulate that the key difference in these normalization methods lies in the effectiveness of the method to distinguish invariant genes from the ones that have altered expression. Therefore, NVSA's unique invariant gene selection approach may be useful for improving the robustness of alternative methods.

**Figure 3:**
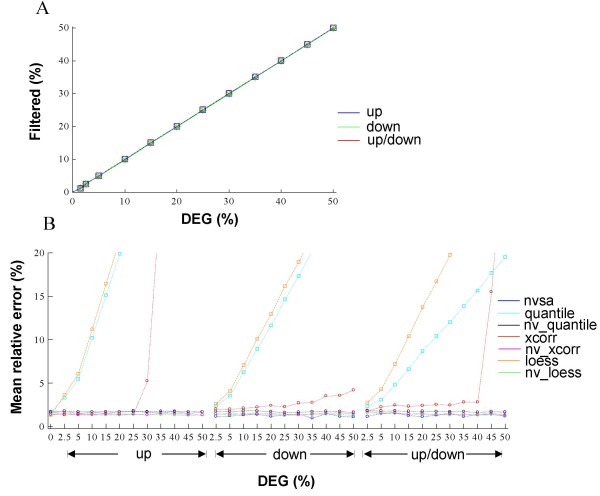
**Use of NVSA for improving normalization accuracy**. The NVSA variable selection function (denoted as nv) was used prior to quantile, lowess, or xcorr normalization analysis of Dnl data series. (*A*) The percentage of variant genes filtered out by the NVSA variable selection procedure increases along with the percentage of gene effects. (*B*) Normalization accuracy measured by the mean relative error versus the total percentage of gene effects represented by DEG. Up, down, up/down indicate the gene effects as up-, down-, and an equal mixture of both, respectively. Errors that are greater than 20% are not shown to allow a better view of details in the low error region.

Using the NVSA method, we filtered out the genes whose log_2 _expression ratios (LER) are outside of the invariant gene modes (named variant genes). The percentage of genes that have been filtered out correlates well with the percentage of gene effects (Fig. 3A). Normalization by lowess, quantile, and cross-correlation on the remaining data are subsequently carried out under the same default conditions as used previously. It should be noted that the NVSA filtering plus lowess analysis is equivalent to replacing the rank invariant step of the invariant set method with the NVSA variable selection procedure. Thus, normalization by the curve-fitting component (lowess procedure) of the invariant set method was not necessary and was not performed. To prevent errors caused by extrapolation, only the normalization errors on the invariant genes were estimated. As expected, the accuracy of normalization has been substantially improved with the introduction of NVSA analysis prior to data normalization (Fig. 3B), as compared to the one without, for all of the alternative methods tested. In most cases, NVSA-assisted alternative methods perform as well as the full NVSA application. The results indicate that the performance gain observed in Figure 2 is largely due to the variable selection step of NVSA, and NVSA is most effective for identifying a set of invariant genes that can be used to produce an accurate normalization procedure.

### Gene effects result in skewed distributions

**Figure 4:**
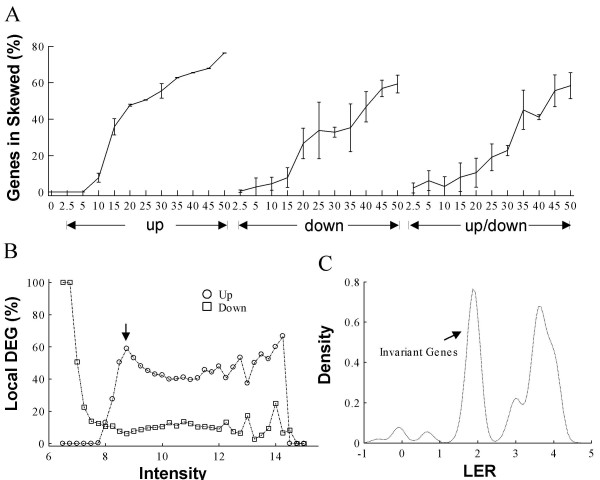
**Positive correlation between the percentages of skewed LER distribution and gene effects**. (*A*) Percentage of genes in skewed local LER distributions along with gene effects for the Dnl data series. The x-axis is the same as in Figure 2. The error bar represents standard deviation computed from 3 replicates. (*B*) Actual local DEG percentage at each intensity interval. Data used is Dnl simulated with 50% gene effects with an equal proportion of up- and down- regulated genes globally. Black arrow points to the intensity interval used in C. Intensity refers to geometric mean intensity before normalization, which is known as A of a MA plot. (*C*) Probability density distribution of LER in the interval highlighted in B. LER: logarithmic expression ratio.

Normalization methods assuming symmetric gene effects are expected to be sensitive to skewed LER distributions, a condition that can occur when there is an over abundance of up- or down-regulated genes within a small range of intensities in a dataset. Using the simulation data, we measured the extent of the skewed LER distribution within each bin according to the AG method in the Dnl data series [21]. The studies reveal that the percentage of genes in skewed distributions increases concurrently with an increased percentage of gene effects (Fig. 4A). Studies of actual local DEG percentages reveal uneven distribution of up- and down-regulated genes locally, when these two populations are balanced globally (Fig. 4B, C). Thus, both globally- symmetric and asymmetric gene effects cause a local skewing of the LER distributions and these observations are consistent with our performance assessment results.

### Agreement of normalization regardless of gene effects

Normalization methods that are robust to gene effects are expected to output similar normalization curves from data including or excluding genes containing gene effects. We therefore use this criterion to compare the performance of normalization methods on experimental microarrays where the true normalization factors are unknown but the genes that are differentially expressed are known. The Golden Spike data set, developed by Choe et al. [14], is unique in that the relative concentrations of all 3,866 gene transcripts between control (C) and spike-in (S) samples are defined: 2,535 have identical concentrations between S and C samples; 1,331 have increased concentration in S over C samples at a fold-change level ranging from 1.2 ~ 4 folds. Thus, the dataset has approximately 34% gene effects between C and S samples yet 0% gene effects between replicate arrays. This allows us to study the agreement of normalization under the conditions of large gene effects versus no gene effects, as well as between the analyses from all genes versus non-DEG only.

**Figure 5:**
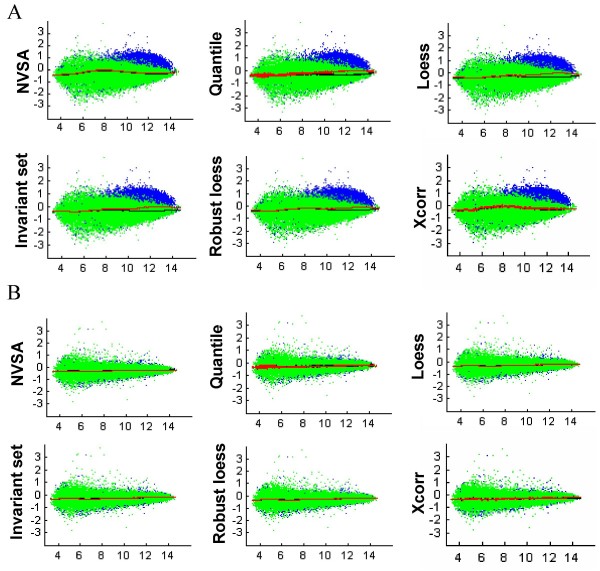
**Agreement of normalization between all data and a subset of data**. Normalizations were carried out from perfect-match probes of all probes (blue) or a subset of probes (green) that are spiked in to be unaltered at transcript levels between C and S samples of Golden Spike data. Fitted normalization curves over all (red) or the subset (black) data are shown in MA plots of non-replicate arrays (*A*) and replicate arrays (*B*). Microarrays used in A are C2 as baseline array vs. S2. Data used in B are C2 as baseline array vs. C1. There are approximately 34% gene effects in non-replicate arrays. C1, C2: C chip replicate 1, 2, respectively. S2: S chip replicate 2. It should be noted that the two normalization curves generated by Xcorr in A are not significantly different in this non-replicate array pair as defined in Fig. 6.

**Figure 6:**
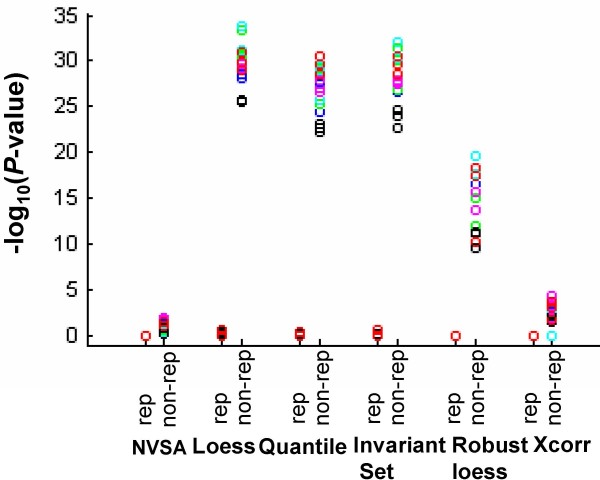
**Kolmogorov-Smirnov tests for agreement of normalization in the analysis of Golden Spike data**. Assessment of statistical difference (Kolmogorov-Smirnov tests) between normalized intensities computed via all-gene approach and normalized intensities via non-DEG genes with the same normalization method. All normalized intensities were calculated relative to a reference array. Each one of six arrays was chosen as a reference array. A total of 12 replicate array pairs and 18 non-replicate arrays pairs could be formed. Blue, green, cyan, magenta, black, and red mark the reference array C1, C2, C3, S1, S2, and S3, respectively. Difference in two normalization curves is statistically significant when *P *value < 0.01.

Figure 5 illustrates typical agreement of normalization curves computed from all genes (red) and non-DEG (black) when C and S chips (non-replicate; Fig. 5A) or C and C chips (replicate; Fig. 5B) are paired. The red and black lines overlap over the entire intensity range in all methods with the replicate array pair, indicating that normalized intensities from all genes agree with the ones computed from non-DEG genes under 0% gene effects (Fig. 5B). However, in the non-replicate array pair, all methods except NVSA and cross-correlation display significant divergence between black and red colored normalization curves, and the divergence appears rising with the increasing amounts of DEG (Fig. 5A). We then employed the two-sample Kolmogorov-Smirnov test statistic to test the null hypothesis that the two sets of normalized intensities (or two normalization curves) were drawn from the same population (Fig. 6). At a 1% significance level (p < 0.01), the null hypothesis is accepted for all NVSA analyses under conditions of both replicate and non-replicate array pairs, indicating that the normalized intensities based on all genes by NVSA are statistically indistinguishable from the ones based on known invariant genes on all tested conditions. However, quantile, lowess, invariant set, and robust lowess methods display statistically significant divergence in two populations of normalized intensities on all non-replicate array pairs (Fig. 6), yet no significant divergence on replicate pairs. In data analyzed by the cross-correlation method, 11 out of 15 non-replicate array pairs display statistically significant divergence, this agrees with our previous observation with simulation data that the method generates substantial error rates when gene effects are greater than 30%.

Therefore under our test conditions, NVSA is most robust for normalizing experimental microarrays containing significantly large and asymmetric gene effects. The performance characteristics associated with NVSA could be important since it removes the requirement for a priori knowledge of or assumptions about housekeeping genes in experiments involving multiple tissues or stages of development in which it is reasonable to assume the existence of a large number of DEG.

## Discussion

In the current study, we describe a novel two-dimensional nonparametric normalization method to address the problem of significant gene effects in Affymetrix microarrays. This problem is significant in experiments involving multiple tissues or developmental stages since knowledge of and assumptions about gene expression, including those of housekeeping genes are tenuous. We demonstrate the high performance characteristics of our new analysis method in dealing with the influence of gene effects during normalization.

We show that the NVSA approach maintains high accuracy and high precision during the normalization of data containing 0 to 50% simulated gene effects. Normalization by alternative approaches, however, becomes increasingly compromised when the extent of gene effects increases. The analysis on experimental microarrays confirms that NVSA performs better than alternatives when gene effects are large. These performance characteristics are consistent with the difference in model assumptions and method approaches. NVSA is nonparametric for both the intensity and LER dimensions, as opposed to the quantile, lowess, and invariant set methods that are nonparametric in one dimension (intensity). Nonparametric versus parametric assumptions of LER distribution is the key distinction between NVSA and the available alternatives in model assumptions. Nonparametric assumptions of LER distribution means that the algorithm does not assume the symmetry, modality of LER distributions. When LER is symmetrically distributed, all methods are expected to perform well and our results confirm this. When LER is asymmetrically distributed, nonparametric treatment of LER distribution for identifying the center of invariant genes is expected to be superior to parametric methods, since NVSA characterizes LER distributions rather than assuming them. The variable approximation process of NVSA is functionally similar to other nonlinear normalization methods including lowess. However, the dimensions of the input data (# bins + 1) are dramatically reduced (usually < 100 data points for NVSA compared with all array data points in lowess), resulting in a faster implementation (an average of 12, 25, 300, and 600- fold faster than invariant set, lowess, robust lowess, and cross-correlation methods, respectively).

Although both NVSA and the invariant set methods specifically aim to identify invariant genes, the variable selection process of NVSA takes a more sophisticated approach to achieve two aims: 1) to segregate gene and intensity effects in mutually independent ways using a two-dimensional nonparametric approach, and 2) to identify invariant genes even when they are not in the predominant classes using a rule-based global optimization approach. Our study demonstrates that NVSA provides superior performance to the invariant set method under many common and important experimental conditions.

**Figure 7:**
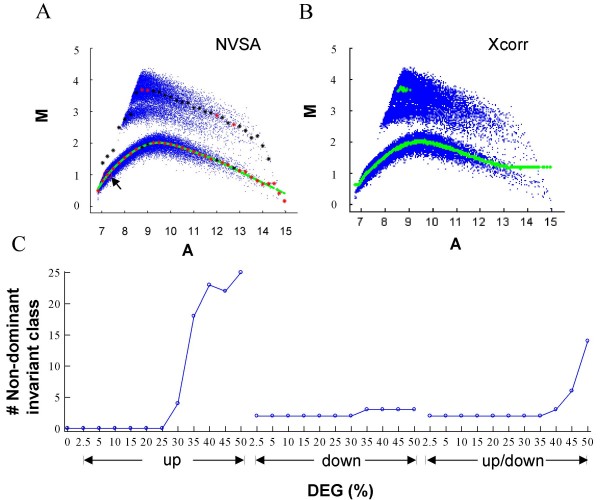
**Effect of non-dominant invariant genes on data normalization**. (*A*) MA plot of baseline array versus Dnl data containing 30% up-regulated genes. Each blue point represents a feature in a microarray. Red and black dots mark the expected values of the 1^st ^and 2^nd ^largest peaks of LER density distribution for each interval by NVSA method, respectively. Green labels each NVSA-fitted normalization value. Black arrow points to the center interval (red circle) of the seed invariant in NVSA analysis. (*B*) Fitted normalization values by cross-correlation method on the same microarray data (Green). (*C*) Number of non-dominant invariant gene classes that occur under each gene effect condition of Dnl data set. Invariant gene class is defined as non-dominant when the class is not the largest peak of LER distribution in NVSA analysis. It is noteworthy that the two or three non-dominant invariant gene classes shown in data containing 2.5 – 50% down- or 2.5 – 40% up/down- regulated genes are all located in the first two or three intensity intervals where normalization errors are not 100% included in the calculation due to the exclusion of boundary data points. Thus in these data, the effect of non-dominant invariant genes may not be reflected in normalization errors.

Cross-correlation is a newly developed normalization to address the issue of asymmetric gene effects. The method accumulates significant amounts normalization error starting from 30% gene effects in simulation data and produces statistically significant disagreement between results of normalization on all genes and those on only invariant genes in the majority of golden spike data containing ~34% gene effects. We speculate that the cross-correlation method may be dependent on an assumption that the local invariant genes are present as a predominant class. We have generated a MA plot for Dnl data containing 30% up-regulated genes, which shows that in four intensity bins, invariant genes are not the predominant classes (Fig. 7A, red dots in variant genes). Consistent with this notion, erroneous normalization by cross-correlation occurs in one of the regions where invariant genes are not dominant (Fig. 7B). We have further plotted the number of non-dominant invariant gene classes along with all gene effects conditions (Fig. 7C). Visual comparison of Fig. 7C to Fig. 2A suggests that the conditions where the cross-correlation method fails are generally the conditions where the number of non-dominant invariant classes is greater than 3. A higher number of non-dominant invariant class correlates with a larger normalization errors in this method. These analyses results support our hypothesis that the cross-correlation method requires an implicit assumption that may be violated in simulation data sets.

Our current results confirm the hypothesis that significant gene effects cause skewed LER distributions, the conditions under which the critical assumptions made by other approaches break down and cause the methods to produce errors. Interestingly, the percentages of genes in skewed distributions are higher in up- than in down-regulated genes. Skewed distributions have also been found in data simulated with globally balanced gene effects. We reason that it is the result of combinations of asymmetric intensity distributions and boundary effects. It is known that the intensity distribution in a typical microarray is non-normal, with a long tail skewed to the right and denser distribution on the left side. The magnitude of negative fold-changes is thus more likely to be subdued by a scanner's low detection limit than the magnitude of positive fold-changes to be tempered by the upper detection limit; Positive fold-changes are thus likely to increase the percentage of local DEG more than negative fold-changes do due to the asymmetric intensity distribution.

When the extent of gene effects is locally greater than 50%, invariant genes are not in the largest peak of local LER distribution. This may cause NVSA to mistake the variant genes as invariants, which may influence the accuracy of seed invariant class selection. When this does occur, NVSA is expected to break and incur large errors. To estimate exactly when the performance of NVSA breaks, we have generated Dnl or D1.25 data containing 55 to 95% gene effects in a 5% increment, and then 96 to 100 percent in a 1% increment. For Dnl data, the NVSA normalization has maintained similar small error rates as seen in Fig. 2 and then start to break when data contains 100% up-, 90% down-, and 85% up/down-regulated genes, respectively [See Figure S2 of additional file 1]. Correspondingly, large normalization errors start to be observed in NVSA-normalized D1.25 data containing 95% up-, 75% down-, and 80% up/down-regulated genes, respectively. In all theses cases, the performance break-down is due to the miss-selection of seed invariant classes. This error may be corrected by manually typing the correct corresponding intensity interval of the seed invariant class if the class is visually definable. Another factor that may influence NVSA performance is the choice of bin width, which can cause overfitting or underfitting of the data. However, some local overfitting or underfitting errors can be mitigated by the following spline smoothing procedure. We set our default bin width at 0.25. Our limited study indicates that small variations in the choice of bin-width do not affect NVSA performance as long as there are sufficient numbers of data points in the bin. When the whole array data points are much smaller than a typical Affymetrix array, a coarse binning may be more appropriate. As with most normalization methods, the performance of NVSA may also be affected by the accuracy of background subtraction. For example, nonzero intensities from large amounts of empty cells may convolute the distribution of invariant genes and thus interfere with the estimation of intensity effects. In addition, various forms of experimental noise may influence the choice and performance of normalization methods. For any given experimental microarray, it is beneficial to validate with statistical tests [22] whether a normalization method is needed and which normalization method is most suitable.

## Conclusion

Our analyses revealed that a high percentage of gene effects, whether they are globally balanced or not, causes local asymmetric distributions of LER. The skewed LER distributions increasingly interfere with the performances of several normalization methods that are widely used today. We presented a novel normalization method NVSA that implements a unique integrated approach for selecting invariant genes. The accuracy in invariant gene selection was achieved using a two-dimensional nonparametric approach for peak identification and a rule-based global optimization method for peak selection. We validated the high performance of the new method on simulated and experimental data sets containing up to 50% gene effects. Our analysis results indicated that the new method may be a useful tool for data normalization analysis in any experiment, and especially in experiments containing samples from different tissues or development stages in which it is reasonable to assume the existence of a high percentage of gene effects.

## Methods

### Construction of simulated data

Ground truth data was constructed where each intensity value is randomly obtained from the perfect-match probes of Affymetrix U95 Latin square dataset [23]. The resulting data has similar intensity distribution to the original and contain a total of 50,504 probes. Gene effects were introduced into the ground truth data by reducing or increasing the values of randomly chosen data points by 2, 3, or 4 folds (these fold changes are present at 1:2:1 ratios) at a specified percentage (0 – 50%). The final simulated dataset (*y*_*i*_) were generated following a multiplicative error model *y*_*i *_= *s*_*i*_*u*_*i *_+ *ε*_*i*_, where *u*_*i *_is the simulated true expression value for *i*th *gene *and *s*_*i *_is the scaling factor. The error *ε*_*i *_is normally distributed with a mean of zero and standard deviation of *cs*_*i*_*u*_*i*_, where *c *= 0.1. Each percentage has three simulated replicates. Data are floored to the minimal true intensity, and ceiled to the largest value of unsigned16-bit data.

### Golden Spike data

The 6-chip Golden Spike dataset consists of 3 replicate control chips and 3 replicate sample chips generated by Choe et al. [14]. Briefly, cDNA clones corresponding to 3,866 unique probe sets were mixed to the extent that the relative concentration of 2,535 transcripts are constant between control and samples, whereas the rest of clones have increased concentration in samples, causing ~34% gene effects. Most of the increases in concentration are <= 1.5 fold.

### Microarray data analysis

All analyses were conducted in MatLab [19] under default settings. Cross-correlation version 2 was generously provided by the authors [20]. All other alternative normalization methods are supplied by the bioinformatics toolbox in MatLab R2008a. Normalization were performed on all perfect-match probes when applicable, except in Golden Spike data, the intensity values from empty spots (10,144 probe sets) were all excluded from normalization analysis. This is due to the observation that large amounts of empty spots contain significant intensities after background subtraction, which interferes with all normalization methods under current study. In the analysis of Golden Spike data, background subtraction was performed using maximum likelihood procedure in MatLab [19] prior to normalization. To avoid introducing bias, data points that are floored or ceiled during simulation data generation are excluded from the calculation of normalization errors, coefficient of variation, and the percentages of genes in skewed LER distributions.

## Authors' contributions

TTN developed the NVSA method and performed all the analyses. All authors conceived and designed the experiments and drafted the manuscript. All authors read and approved the final manuscript.

## Supplementary Material

Additional file 1 **Additional data analysis results**. figures illustrating accuracy of normalization in the analysis of linear simulated data and gene effect conditions before and at the NVSA performance break-down. Click here for file
