# Supplementary material for: Use of normalization methods for analysis of microarrays containing a high degree of gene effects
Source: BMC Bioinformatics. 2008 Nov 28;9:505. doi: 10.1186/1471-2105-9-505 (PMC2612699; doi:10.1186/1471-2105-9-505)
Supplement: Additional file 1 — Additional data analysis results. figures illustrating accuracy of normalization in the analysis of linear simulated data and gene effect conditions before and at the NVSA performance break-down. [file 1471-2105-9-505-S1.pdf]

Figure S1. Accuracy of normalization in the analysis of linear simulated data

Mean relative error in normalization (scaling) factors determined by the seven different normalization methods using D1.25 dataset. Errors that are greater than 50% are not shown. The x-axis corresponds to the total percentage of gene effects (shown as DEG) in a microarray, and up, down, and up/down indicates these effects as up-, down-, and an equal mixture of both, respectively. Median: global median normalization.

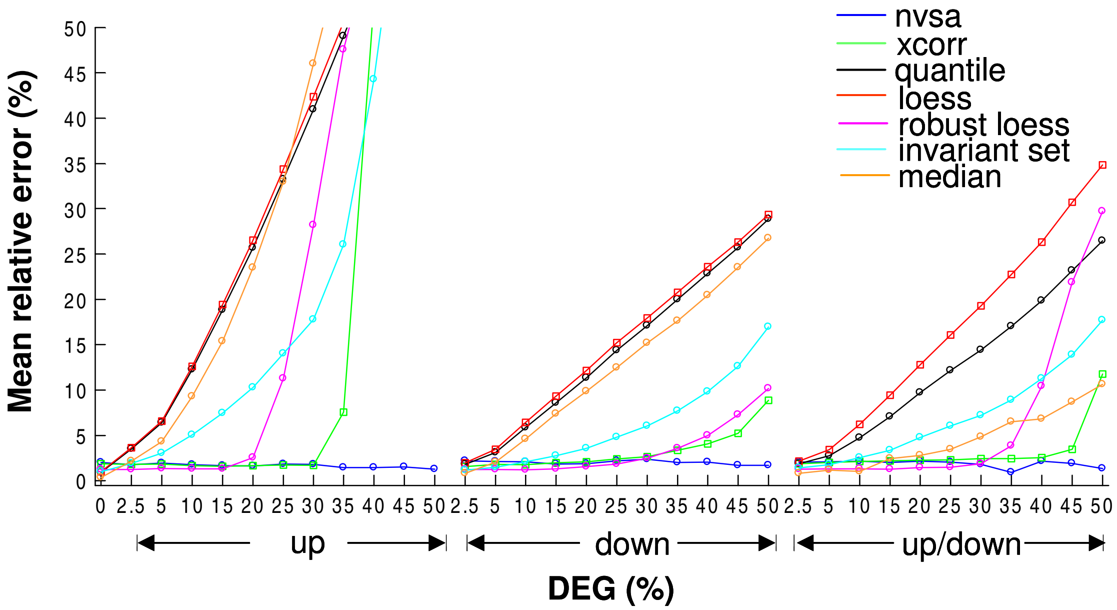

Figure S2. NVSA normalization before and at the condition of performance break-down

MA plots of NVSA normalization using Dnl data. Gene effect conditions before (left) and at (right) the performance break-down are shown. Each blue point represents a feature in a microarray. Red and black dots mark the expected values of the 1st and 2nd largest peaks of LER density distribution for each interval by NVSA method, respectively. Green labels each NVSA-fitted normalization value. Each red circle labels the center interval of the seed invariant in NVSA analysis.

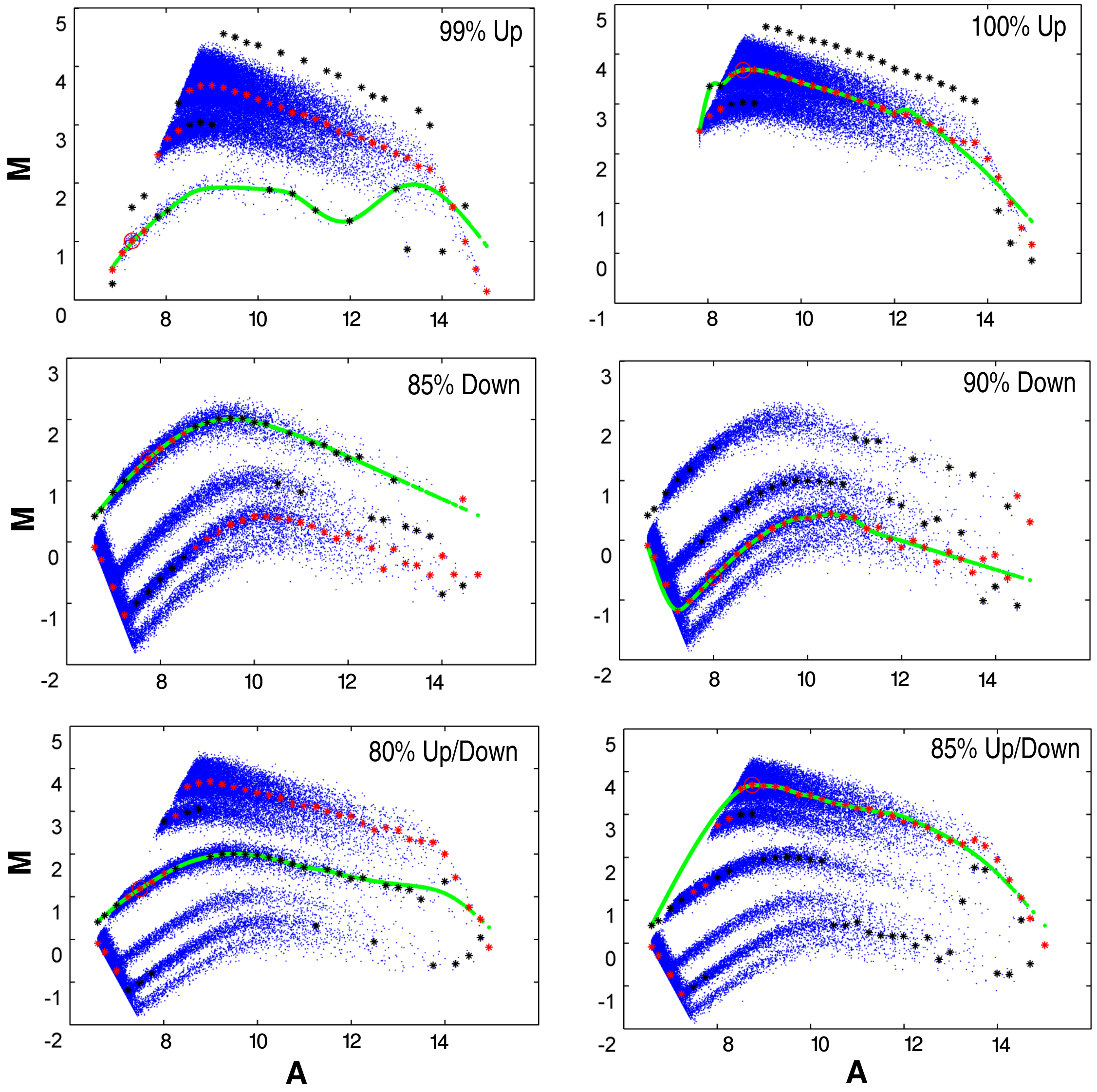
99% Up
100% Up
85% Down
90% Down
80% Up/Down
85% Up/Down
M
A
